# Ureteral metastasis in colorectal cancer: A case report and review of literature

**DOI:** 10.1016/j.eucr.2021.101851

**Published:** 2021-09-13

**Authors:** Justin Loloi, Kevin Kuan, Jinrong Cheng, Evan Kovac, Ahmed Aboumohamed

**Affiliations:** a Department of Urology, Montefiore Medical Center, Albert Einstein College of Medicine, Bronx, NY, 11061, USA; b Department of Pathology, Montefiore Medical Center, Albert Einstein College of Medicine, Bronx, NY, 11061, USA

**Keywords:** Ureter, Metastasis, Colorectal, Urologist, Hydronephrosis

## Abstract

Colorectal cancer (CRC) is a common clinical entity. A significant proportion of patients experience metastatic disease, typically in the form of lung and liver spread. We present the case of a CRC cancer patient with distant metastasis to the ureter, causing hydronephrosis. Ureteroscopy and biopsy confirmed the diagnosis and the patient was subsequently treated with percutaneous nephrostomy tube placement. Distant spread of CRC to the ureter represents an exceedingly rare phenomenon. This case highlights the importance of heightened index of suspicion for ureteral involvement in CRC when hydronephrosis is identified on staging or surveillance cross sectional imaging.

## 1 Introduction

Colorectal cancer (CRC) is a major cause of morbidity and mortality, accounting for more than 9% of cancers worldwide.^1^ Survival is highly dependent on stage of disease at diagnosis, with a particularly poor prognosis with more advanced stages of the disease.^1^

In patients with CRC, metastasis is the primary driver of cancer-related mortality. Interestingly, the specific site of metastasis is associated with cancer-specific survival, and may serve as an independent prognostic indicator in metastatic CRC.^2^

Distant genitourinary metastasis in CRC is sparsely reported in the literature. Specifically, to our knowledge, CRC cancer with metastasis to the ureter is an exceedingly rare entity. We present a case of rectosigmoid cancer with metastasis to the ureter.

## 2 Case presentation

A 57 year-old male with a past medical history of HIV (diagnosed 17 years prior to presentation, CD4 > 600, viral load undetectable on antiretroviral therapy) and sigmoid moderately differentiated adenocarcinoma undergoing neoadjuvant chemoradiation with capecitabine presented to Urology clinic after a computed tomography (CT) abdomen/pelvis with contrast revealed moderate right-sided hydronephrosis with hydroureter down to the mid-distal ureter. No stones were found. At his cancer diagnosis, positron emission tomography (PET)-CT scan showed mildly FDG-avid mesenteric nodes concerning for disease, but no other sites of concern. At the time of his presentation to urology, the patient was asymptomatic without flank pain, lower urinary tract symptoms, or hematuria. In the setting of a creatinine of 1.9 mg/dL (baseline 1 mg/dL), it was recommended he undergo right ureteroscopy and retrograde pyelogram with possible right ureteral stent placement.

**Fig. 1:**
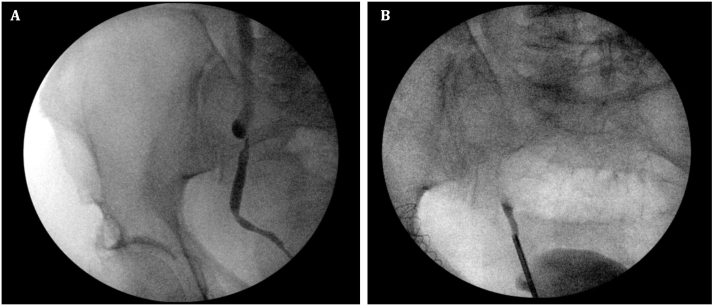
**(A)** Retrograde pyelogram showing an area of narrowing in the mid-distal right ureter, **(B)** Subsequent retrograde pyelogram with complete arrest of contrast at the distal ureter.

Right retrograde pyelogram (Fig. 1A and B) revealed an area of narrowing in the right mid-ureter (Fig. 1A). Right ureteroscopy revealed a narrow mid-ureteral lumen with intact mucosa and no intraluminal lesions, suggesting extrinsic compression. The ureteroscope could not traverse the area of narrowing and thus a ureteral stent was placed. Serum creatinine improved to 1.1 mg/dL post-operatively.

Following neoadjuvant chemotherapy, the patient underwent uncomplicated laparoscopic sigmoidectomy and diverting loop ileostomy. Surgical pathology revealed pT3N1b invasive adenocarcinoma of the rectosigmoid colon with negative margins. The ureteral stent was removed post-operatively and subsequent renal bladder ultrasound showed no hydronephrosis bilaterally. The stent was removed since it was assumed the hydronephrosis was secondary to extrinsic compression and there were no intraluminal lesions on CT scan or retrograde pyelography.

**Fig. 2:**
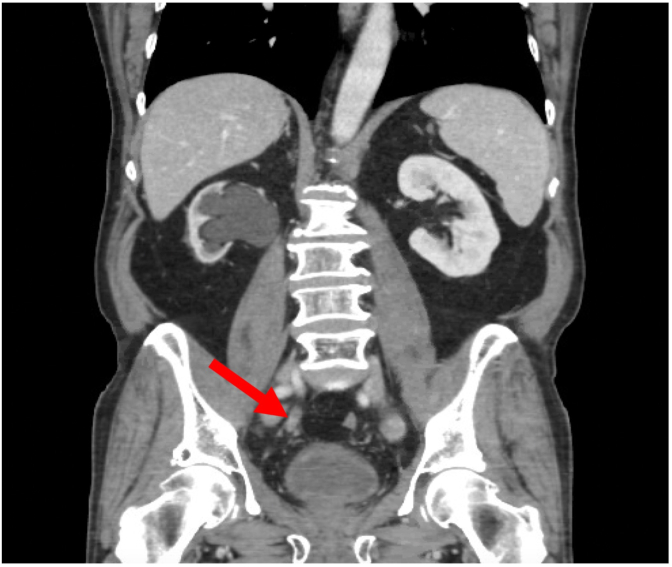
CT abdomen/pelvis revealing marked right hydronephrosis secondary to an endoluminal lesion in the distal ureter.

One year later, surveillance CT chest/abdomen/pelvis showed several new mesenteric soft tissue nodules concerning for peritoneal carcinomatosis and new interval development of right renal atrophy secondary to marked right hydroureteronephrosis, secondary to an endoluminal soft tissue density lesion in the right distal ureter, concerning for possible urothelial tumor (Fig. 2). Serum creatinine rose to 1.8 mg/dL.

**Fig. 3:**
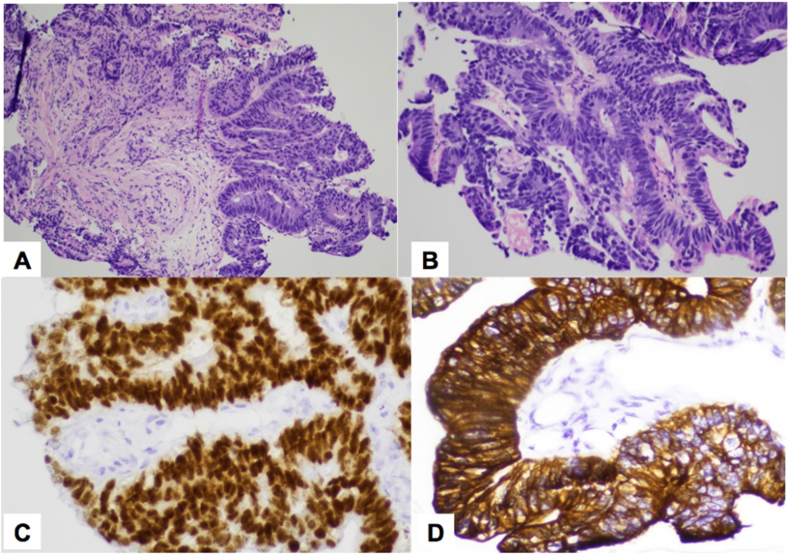
(**A)** and (**B)**, Sections of the ureter tumor showed moderately-differentiated adenocarcinoma with crowded, irregular glandular structures. The neoplastic glands were formed by tall and columnar epithelial cells with nuclear hyperchromasia and loss of nuclear polarity. **(C)** and (**D)**, Immunohistochemical stains showed that the tumor cells were diffusely positive for CDX-2 (C) and CK20 (D).

On right retrograde pyelogram, only 2 cm of distal ureter opacified, and the remainder of the collecting system was unable to be filled with contrast (Fig. 1B). Semirigid right ureteroscopy identified a sessile mass completely obstructing the lumen in the distal right ureter. We were unable to place a stent. Multiple ureteroscopic biopsies of the mass were taken and pathological examination revealed adenocarcinoma, favoring a colorectal primary (Fig. 3). The immunoprofile of the ureter tumor was therefore consistent with a carcinoma of colorectal origin. Next-Generation Sequencing analysis demonstrated a similar molecular fingerprint between the colon resection and the ureteral biopsy specimens. The molecular study results further confirmed the colorectal origin of the ureter tumor.

In the setting of persistent hydronephrosis, the patient had a right percutaneous nephrostomy tube placed with subsequent CT abdomen/pelvis revealing an atrophic right kidney with resolution of the hydronephrosis. Due to widespread metastatic disease burden, localized resection of the right ureteral metastasis was not considered. The patient continues to receive routine nephrostomy tube changes every 3 months and systemic therapy for his metastatic disease.

## 3 Discussion

Although a considerable number of CRC patients develop distant metastases,^3^ metastasis to the ureter is an exceedingly rare event.

In their systematic review of ureteral involvement by metastatic malignant disease, Hu et al. identified 265 cases with various cancer metastasis to the ureter. The most common sites of primary tumor included the prostate, bladder, breast, gut, and lymphoma. Gut cancer (small and large bowel) metastasis to the ureter comprised a small portion of the described cases (35/265 cases). Treatment of these cases consisted primarily of renal decompression without metastectomy and segmental ureterectomy with curative intent in a minority of cases.^4^ In this analysis, metastatic ureteral cancer from an intestinal primary was diagnosed at an average of 24 months after diagnosis of the primary lesion. In the present case, the patient was found to have hydronephrosis one month after his primary cancer diagnosis. Thus, our case displays the relatively early potential for ureteral involvement.

Even when asymptomatic, untreated obstructive may result in renal injury and fibrosis.^5^ Given the rarity of ureteral metastasis in CRC, urologic symptoms (if present) such as lower urinary tract symptoms or obstructive uropathy may be mistakenly credited to an unrelated pathologic process. Consistent with national guidelines, it is crucial CRC patients undergo routine surveillance so such metastases can be identified early before the onset of irreparable parenchymal damage. As it pertains to our case, surgeons should then reserve a high index of suspicion for ureteral metastasis when hydronephrosis is identified during follow up.

## 4 Conclusion

In the present case, we report early ureteral metastasis in a patient with colorectal cancer. The patient was incidentally found to have hydronephrosis in the workup for his primary lesion with an initial ureteroscopy unremarkable for an intraluminal lesion. After neoadjuvant chemotherapy and sigmoidectomy, surveillance imaging revealed marked right hydronephrosis and a new endoluminal soft tissue density. Diagnostic ureteroscopy revealed a urothelial mass completely obstructing the lumen in the distal right ureter. Biopsy confirmed the ureteral tumor was consistent with a carcinoma of colorectal origin. Our case highlights the importance of heightened index of suspicion for ureteral involvement in CRC when hydronephrosis is identified on staging or surveillance cross sectional imaging, as the resulting obstructive uropathy can result in diminished renal function and may adversely affect prognosis. Treatment of ureteral metastases should be based on patient's individual stage and prognosis.

## Contributorship statement

JL, KK, JC, EK, AA prepared the manuscript and figures. AA managed the patient and supervised the manuscript preparation. All authors approved the submitted manuscript.

## Funding

None.

## Declaration of competing interest

The authors have no conflicts of interest to disclose.
